# HIV Treatment in a Conflict Setting: Outcomes and Experiences from Bukavu, Democratic Republic of the Congo

**DOI:** 10.1371/journal.pmed.0040129

**Published:** 2007-05-29

**Authors:** Heather Culbert, David Tu, Daniel P O'Brien, Tom Ellman, Clair Mills, Nathan Ford, Tina Amisi, Keith Chan, Sarah Venis, Médecins Sans Frontières

## Abstract

Providing HIV care in conflict settings involves additional obstacles to those generally encountered in other resource-limited settings, say Heather Culbert and colleagues.

## Introduction

Armed conflict and HIV infection have had a profound impact on the societies of sub-Saharan Africa. The number of countries engaged in armed conflict has fluctuated, totalling 24 African states in 2004 [1]; most of these conflicts are intrastate and chronic. The region also bears the world's highest burden of HIV, with more than 25 million people reported to be infected [2].

Although the inter-relationship between HIV and conflict is increasingly clear [3–6], the effect of conflict and instability on the incidence and prevalence of HIV can be unpredictable [6]. Much of the evidence on risks is unreliable, and conflict may both protect a population from HIV by isolating communities from the spread of infection, and increase an individual's risk through displacement, sexual violence, and breakdown of communities and health-care institutions.

Despite an often significant burden of HIV-related mortality and morbidity, few HIV care programmes have been attempted in conflict regions. There is a perception that it will be too difficult to perform safely and effectively, and that HIV prevention and treatment are of secondary importance to concerns such as food, shelter, water and sanitation, basic medical care, and personal security. As outlined in Table 1, providing HIV care in conflict settings involves additional obstacles to those generally encountered in other resource-limited settings.

**Table 1:**
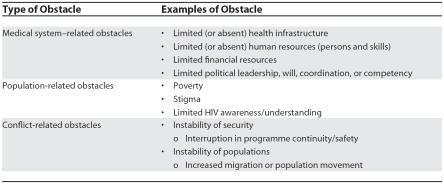
Obstacles to Providing HIV Care in Resource-Limited Chronic Conflict Settings

This paper describes lessons from three years' experience of providing HIV care, including antiretroviral therapy (ART), to a conflict-affected population in the Democratic Republic of the Congo (DRC).

## Chronic Conflict in the Bukavu Region

**Figure 1:**
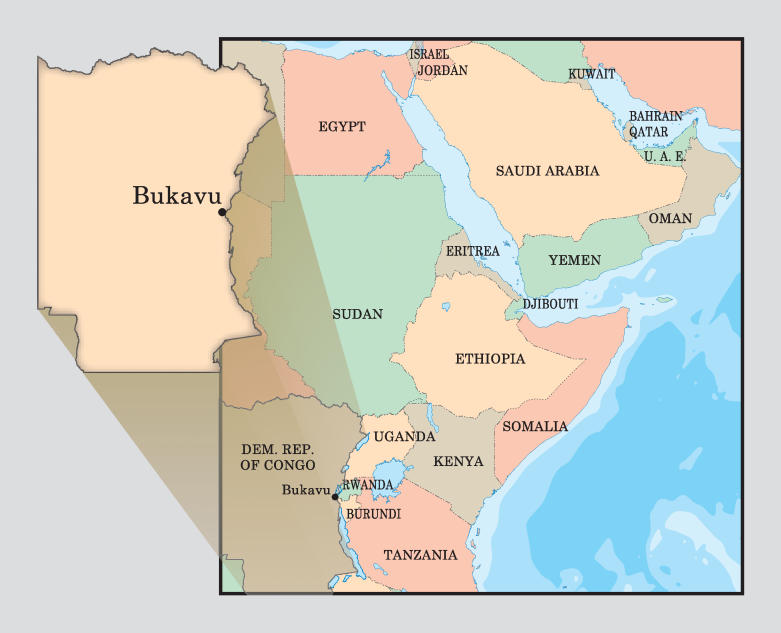
Map of Bukavu, South Kivu, DRC

Bukavu is a city of 600,000 inhabitants located on the southern shore of Lake Kivu in eastern DRC, bordering Rwanda (Figure 1). The region has experienced chronic conflict since 1996, involving the neighbouring states of Rwanda, Uganda, and Burundi, as well as numerous internal guerrilla armies. The conflict in DRC is estimated to have resulted in more than 3.9 million deaths between 1998 and 2004 [7]. Despite a 2001 peace accord and elections held in July 2006, peace in the region remains elusive [8].

## Design of the Médecins Sans Frontières HIV Programme in Bukavu

By the late 1990s, HIV prevalence in Bukavu was estimated at 4%–9% [9]. The Médecins Sans Frontières (MSF) project was initiated in 2000 to provide treatment for those infected with HIV and to help reduce the rate of HIV transmission. Bukavu was chosen for this project because it had a high HIV prevalence, and was an area of relative calm in a region of conflict.

The programme began with treatment of sexually transmitted infections and HIV prevention activities focusing on outreach education and condom promotion for high-risk groups: commercial sex workers, taxi/truck drivers, military personnel, and youth. These activities were conducted in partnership with the Ministry of Health and local non-governmental organisations. In 2002, the first of three voluntary counselling and testing (VCT) sites and the first of two HIV clinics opened. HIV testing was conducted using two parallel rapid diagnostic tests, initially with nurses and other para-medical staff, and later with lay persons, trained as VCT and adherence counsellors. The clinics provided free-of-charge basic primary medical care, prophylaxis and treatment for opportunistic infections including tuberculosis, prevention of mother-to-child transmission of HIV, post-exposure prophylaxis for victims of sexual violence, nutritional support, and inpatient and home-based care. In October 2003, the programme began providing ART—the first service to do so free of charge in eastern DRC.

Most patients were started on generic fixed-dose combination ART with stavudine, lamivudine, and nevirapine. Selection criteria were based on 2003 World Health Organization guidelines [10], and all those who were eligible and consented to treatment received ART. All patients presenting to the clinic were eligible for the programme, including those from as far as neighbouring Rwanda. Before starting ART, patients attended three workshops (usually in groups of 10–20) on HIV and how ART medications work, common side effects and how to overcome them, the importance of adherence, and drug resistance. Nurses, VCT counsellors, physicians, and experienced patients jointly contributed to the adherence sessions. More recently, patients also receive specific education related to planning for episodes of insecurity (described below); they are also educated about what to do if active conflict causes a disruption to the programme. Regular group adherence workshops continue after commencement of ART.

Each clinic is staffed by two physicians, four nurses, and several lay support workers; the physician to patient ratio is currently 1:500, and continues to rise. As such, physician responsibilities, including the initiation and management of ART, are increasingly delegated to nurses. The clinic is supported by a central laboratory with automated haematology, biochemistry, and CD4 cell count capacity.

## Programme Outcomes

**Figure 2:**
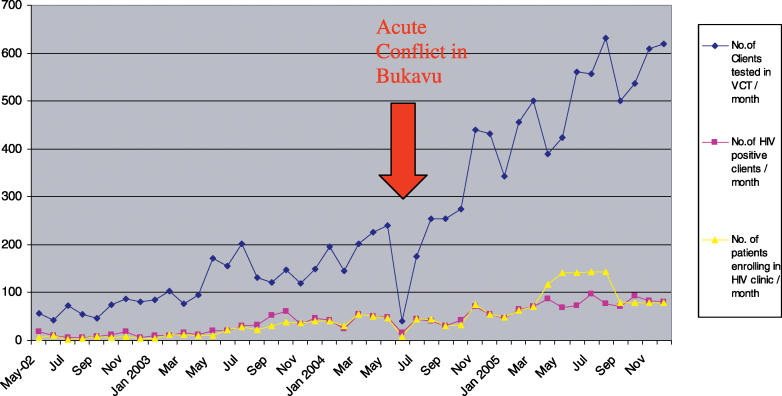
Monthly Number of People Undergoing VCT, Testing HIV Positive, and Enrolling in the HIV Clinic

Between May 2002 and January 2006, 11,076 people had received VCT, of whom 19% were HIV positive. Of those who tested HIV positive, 94% (1,868 patients) received follow-up care in the HIV clinics (Figure 2).

By January 2006, 494 (26%) patients had commenced ART. Of these, 66% were female with a median age of 37 years (interquartile range [IQR] 30–43) and median weight of 51 kg (IQR 44–58). At baseline, 3% were in clinical stage I of HIV (as defined by the World Health Organization's disease staging system), 12% in stage II, 49% in stage III, and 34% in stage IV; baseline median CD4 count was 123 cells/mm3 (IQR 57–195). Good ART adherence, defined as missing less than 5% of pills at last clinic visit and measured by pill counts, was achieved by 99% of patients.

Table 2 shows the outcomes on ART compared to other cohorts in stable resource-limited settings (ART-LINC) and resource-rich settings (ART-CC) [11]. The ART-LINC cohort included patients from 18 ART programmes in Africa, Asia, and South America, and the ART-CC cohort included patients from 12 ART programmes in Europe and North America. CD4 gains at six months were similar in all three groups, and the 12-month mortality and loss to follow-up rates compare favourably to the stable ART resource-poor programmes represented in the ART-LINC cohort. Adherence rate, weight gain, CD4 gain, mortality, and loss to follow-up rates all compare favourably to other reported HIV treatment programmes in non-conflict, resource-limited settings [11–16].

**Table 2:**
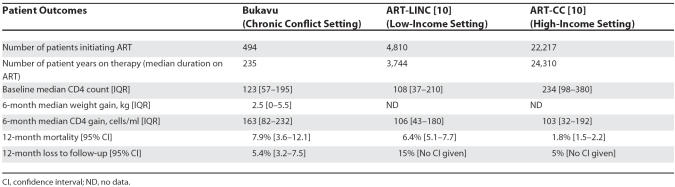
Comparison of ART Patient Outcomes from Programmes in Chronic Conflict Settings with Non-Conflict Settings in Low-Income and High-Income Settings

## Acute Conflict in the City of Bukavu (May–June 2004)

Most armed conflicts in this region have occurred outside Bukavu. However, on May 26, 2004, rebel forces from within the Congolese military invaded the city, resulting in intense fighting for 13 days. During this period hundreds of civilians were killed, thousands fled across the border into Rwanda, and many women were raped [17].

During the crisis, movement of staff and patients was curtailed, and all expatriate MSF staff were evacuated from the city. Basic HIV clinic and hospital functions were maintained during most of the conflict period by a Congolese nurse who lived nearby, although the clinic remained inaccessible to many due to distance and insecurity. Communication during this time was difficult but continued via a radio broadcast and “word of mouth”. The MSF pharmacy was not looted.

Up until the start of the crisis, 66 patients had initiated ART, and these patients experienced the following outcomes:

- 14 had sufficient medication supplies and did not need to come to the clinic;
- three were hospitalised and had ready access to medication;
- 41 came for their antiretroviral (ARV) drugs during the crisis period;
- three were able to collect their ARVs in Rwanda in collaboration with another MSF mission;
- five (of whom four were displaced from Bukavu) had a significant interruption in their ART.

All eventually re-established contact with the clinic and returned to treatment.

Eight months after the conflict, a focus group was held involving five of the affected patients and two nursing staff. They were selected using a stratified simple sampling method to represent the experiences of being displaced or not displaced, with or without treatment interruption. Patients reported physical and mental hardship, illness, geographic displacement, limitations in movement, and the threat of violence and food scarcity. All patients firmly believed that their lives depended on remaining adherent to their ART (Box 1).

**Box 1. Patient Testimony**

**Patient 1**

*Followed in the clinic since 2002, on ART since December 2003. Lives in Rwanda.*

When the fighting started, I thought that it was the end of our lives. I had only treatment for three days and five days of security. I had interrupted my treatment for two days when another patient arrived with the treatments sent by MSF. I was afraid it was the end of my life because more than just the [lack] of medications, I was very sick and I had to be hospitalised with fever and vomiting. It is very important to have the treatment.

**Patient 2**

*Followed in the clinic since May 2003, and on ART since December 2003.*

I heard gun fire all through the night. When I had only five pills left I lost my appetite and felt desperate…but despite the uncertainty I continued to take my treatment at the correct hour… When I had only one pill left I had the courage to go out and seek some more treatment. I went to see [my] nurse [at her home] who informed me that she would be able to distribute ARVs; with that I had a month's worth of treatment. If we have to give up this treatment we will return to how we were at the start, sick.

## Preparing for Instability: The Importance of Contingency Planning

Our experience has shown us that one of the keys to successful provision of ART in conflict settings is preparation for disruption. Programmes should establish contingency plans to enable staff and patients to react as efficiently as possible should the conflict worsen. Key areas to be considered as part of contingency planning are:

**1. Education.** Patient education must stress the importance of excellent ART adherence—in particular the importance of not reducing drug dosing to make stocks last longer if supplies are threatened. However, patients should understand that a forced ART interruption, if managed appropriately, will usually not result in ART resistance, and they should be educated on how to interrupt treatment if necessary (see below). Patients also need to be informed about contingency plans during periods of acute instability, including the mechanisms for supply of medication and ongoing clinical support.

**2. Human resources capacity.** All medical staff should be well grounded in the basics of HIV management (including ART) so that they can fill any human resources gaps that occur during acute conflict. The goal is to create multi-skilled staff that can keep a basic programme running if physicians or other medical staff are evacuated.

**3. Communication networks.** A pre-established communication network between health staff and patients should be established. This may involve such activities as radio broadcasts, church groups, or posters.

**4. Emergency drug stocks and “washout medications”.** An additional 15–30 days emergency stock of ARVs and prophylactic medications can be given to each patient. They should be brought to the clinic on each visit so that their quantity and expiration dates can be monitored. Additionally, a “washout” drug stock can be supplied. Patients on a regimen containing non-nucleoside reverse transcriptase inhibitors (NNRTIs) can be supplied with a seven-day “washout” course of whichever two nucleoside reverse transcriptase inhibitor drugs they usually take (i.e., lamivudine and zidovudine, or stavudine and lamivudine) which they should take after their regular ART is finished. This will reduce the likelihood of NNRTI resistance during the period of effective NNRTI monotherapy brought about by its long half-life [18].

**5. Secure drug storage.** HIV medications may be a target for looting or vandalism. Drug stocks should be divided and stored securely and discreetly at different locations to minimise the impact of such an event. Stock size at programme levels should be kept to the minimum necessary in very unstable areas.

**6. Decentralisation of care.** Decentralisation of clinical services throughout the treatment area will reduce travel requirements, making it easier and safer for staff and patients to access the clinics in times of insecurity.

**7. Cooperation with HIV treatment facilities in neighbouring regions.** Relationships with other programmes that can provide ART in neighbouring regions should be established. An agreement of mutual support between organisations may be critical for the care of displaced patients or for emergency access to medication in case supplies are stolen or destroyed.

**8. Treatment information cards and duplicate medical records.** All patients should be provided with a personal identification card which lists their antiretroviral medications and other significant medical information such as co-morbidities, medical allergies, and CD4 counts. This documentation is useful if the patient becomes displaced and seeks treatment elsewhere or if the clinic medical records are lost or destroyed as a result of looting. Duplicate medical records should be securely stored separately from the clinic site.

**9. Integration with other services.** A partnership with government health services may help ensure access to authorities and supply chains in an acute conflict situation. Importantly, communication links need to be established with all sides of the conflict. These relationships may also provide some protection from looting in times of conflict. Ideally, the perceived benefits of the programme are such that the community as a whole, including government, civil society, and military, are willing to support it despite the conflict.

## Discussion

The HIV project in Bukavu shows that the provision of comprehensive HIV care, including ART, in chronic conflict settings can be feasible and effective, with early treatment outcomes similar to those in HIV projects in non-conflict settings. However, it should be noted that these results have been achieved with the support and resources of an international nongovernmental organisation (MSF), in an urban setting, with the episode of disruption occurring early in the programme, and thus similar results in conflict settings may not always be possible. Nevertheless, the key elements of contingency planning for care delivery in conflict settings are not resource-intense and we believe they can generally be applied to most care programmes.

While assumptions about instability in DRC have prevented most from attempting to provide HIV treatment, the Bukavu project experienced only one significant episode of disruption over a three-year period. An important insight from our experience is that the label of “chronic conflict” should not be assumed to mean frequent instability. In fact, Bukavu's pattern of conflict characterised by periods of relative calm punctuated by episodes of acute insecurity is quite typical for many African conflicts [19], and many of the lessons learnt in the Bukavu programme are currently being applied by MSF in other conflict-affected settings.

Although episodes of insecurity can destabilise ART programmes, putting patients at increased risk of ART interruption and drug resistance, contingency planning and a managed treatment interruption contingency plan can minimise this risk. Given the uncertainties facing HIV programmes in many resource-limited settings—the risk of ruptures in drug supply due to mismanagement, insufficient funding, or lack of staff—the contingency planning issues discussed here may be relevant to more programmes than just those in conflict-affected settings.

Considering the significant interrelationships between conflict and HIV, it is particularly important to target populations affected by both. Given the high numbers of HIV-positive individuals in many conflict-ridden countries, it is not acceptable to wait until the conflict is over before mounting a response.

## Supporting Information

Text S1 Ethics approval (49 KB DOC).

Alternative Language Text S2 French translation of the article by V. Sizaire (138 KB DOC).

Alternative Language Text S3 Spanish translation of the article by L. Fina (31 KB DOC).

## Abbreviations

ART: antiretroviral therapy
ART-CC: antiretroviral therapy programmes in resource-rich settings
ART-LINC: antiretroviral therapy programmes in resource-poor settings
ARV: antiretroviral
DRC: Democratic Republic of the Congo
IQR: interquartile range
NNRTI: non-nucleoside reverse transcriptase inhibitor
VCT: voluntary counselling and testing
