# Supplementary figures and images for: HIV Treatment in a Conflict Setting: Outcomes and Experiences from Bukavu, Democratic Republic of the Congo
Source: PLoS Med. 2007 May 29;4(5):e129. doi: 10.1371/journal.pmed.0040129 (PMC1880839; doi:10.1371/journal.pmed.0040129)

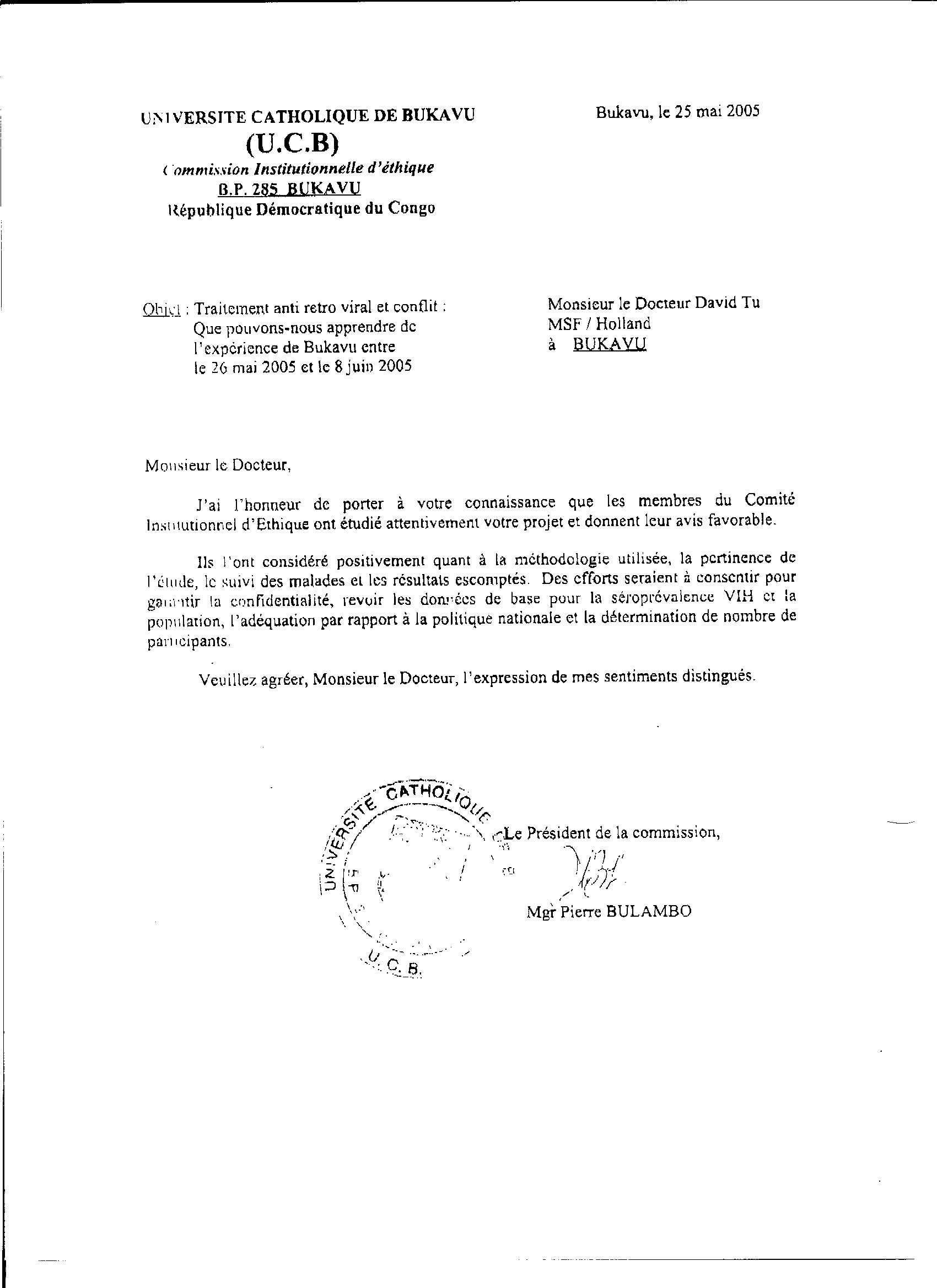

Supplement: Text S1 — (49 KB DOC). [file pmed.0040129.sd001.doc]
