# Supplementary material for: HIV Treatment in a Conflict Setting: Outcomes and Experiences from Bukavu, Democratic Republic of the Congo
Source: PLoS Med. 2007 May 29;4(5):e129. doi: 10.1371/journal.pmed.0040129 (PMC1880839; doi:10.1371/journal.pmed.0040129)
Supplement: Alternative Language Text S2 — (138 KB DOC). [file pmed.0040129.sd002.doc]

HEALTH IN ACTION
Traitement du VIH dans un contexte de guerre: 
Résultats et Expériences à Bukavu, République Démocratique du Congo

Heather Culbert1, David Tu1, Daniel P. O'Brien1, Tom Ellman2, Clair Mills1, Nathan Ford2, Tina Amisi3, Keith Chan4, Sarah Venis2 

1 Medecins Sans Frontieres Hollande, Amsterdam
2 Medecins Sans Frontieres, Grande-Bretagne, Londres
3 Medecins Sans Frontieres Hollande, Bukavu, République Démocratique du Congo
4 British Columbia Center for Excellence for HIV/AIDS, Vancouver, Canada.


Les conflits armés et l'infection du VIH ont eu un impact profond sur les sociétés de l'Afrique sub-Saharienne. Le nombre de pays engagés dans des conflits armés a fluctué, atteignant un nombre total de 24 pays Africains en 2004[1]; la plupart de ces conflits sont internes et chroniques. Cette région est aussi la plus affectée par l'épidémie du VIH, avec plus de 25 millions de personnes infectées.[2] 

Bien que la relation entre le VIH et les conflits apparaisse de plus en plus claire,[3-6] les effets des  conflits et instabilité sur l'incidence et la prévalence du VIH peuvent être imprévisibles.[6] Le conflit peut, à la fois, protéger une population contre le VIH en isolant les communautés de la propagation de l'infection, et augmenter le risque individuel par le déplacement, la violence sexuelle et l'effondrement des communautés et des services de santé. 

Malgré une mortalité et morbidité liées au VIH souvent importantes, peu de projets VIH ont été tentés dans des régions en conflit. Le sentiment est que ces projects seraient d'une part trop difficiles à mettre en place (au niveau de la securite mais aussi de leur efficacite) et d'autre part que la prévention et le traitement du VIH sont secondaires par rapport à la nourriture, le logement, l'eau et l'assainissement, les soins de santés de base et la sécurité du personnel. Comme le montre la Table 1, offrir des services de prise en charge du VIH dans des zones de conflit implique des contraintes supplémentaires à celles généralement rencontrées dans d'autres contextes à ressources limitées.  

Ce papier décrit les leçons apprises pendant 3 ans d'expérience de soins VIH, y compris la thérapie antiretrovirale pour une population affectée par un conflit en République Démocratique du Congo (RDC). 

Conflit chronique dans la région de Bukavu  

Bukavu est une ville de 600 000 habitants située sur le rivage Sud du Lac Kivu à l'Est de la RDC, à la frontière du Rwanda (figure 1). Cette région est en conflit chronique depuis 1996, impliquant les états voisins du Rwanda, Uganda et Burundi, ainsi que de nombreuses guérillas internes. Le conflit en RDC aurait causé plus de 3.9 millions de décès entre 1998 et 2004.[7] Malgré un accord de paix en 2001 et des élections en juillet 2006, la paix dans la région reste aléatoire.[8]

Conception du programme VIH MSF à Bukavu

A la fin des années 90s, la prévalence du VIH à Bukavu était estimée à 5-10%. En 2000, le projet MSF a commencé à offrir traitement aux patients infectés par le VIH et à aider à réduire le taux de transmission. Bukavu a été choisi, pour ce projet, en raison de sa prévalence VIH élevée et de son calme relatif dans cette région en conflit.

Le programme a commencé avec le traitement des maladies sexuellement transmissibles et des activités de prévention du VIH se concentrant sur l'éducation des populations et la promotion du préservatif pour les groupes à risque : prostituées, conducteurs de taxi ou camion, militaires et jeunes. Ces activités étaient menées en collaboration avec le Ministère de la santé et des organisations non gouvernementales locales. En 2002, les trois premiers centres de dépistage volontaire (CDV) et les deux premières cliniques VIH ont ouverts. Le test VIH était assuré par 2 tests rapides faits en parallèle, et l'accompagnement des patients était assuré, au début, par des infirmières et du staff para-médical, ensuite, par des volontaires formés au dépistage volontaire, à l'accompagnement et à l'adhérence. Les cliniques offraient des soins médicaux primaires gratuits,  prophylaxie et traitement des infections opportunistes, y compris la tuberculose, prévention de la transmission mère à l'enfant du VIH, prophylaxie après exposition aux victimes de violence sexuelle, support nutritionnel, et soins hospitaliers et à domicile. En octobre 2003, le programme a commencé le traitement anti-retroviral – le premier service gratuit en RDC de l'Est. 

La plupart des patients a commencé avec des combinaisons génériques d'ARV comprenant la Stavudine, la Lamivudine et la Nevirapine. Les critères de sélection étaient basés sur le guide de l'OMS 2003,[9] et tous les patients éligibles et consentant reçurent le traitement ARV. Tout patient se présentant à la clinique étai éligible pour le programme, y compris ceux originaires du Rwanda. Avant de commencer le traitement ARV, les patients ont participe à 3 sessions (habituellement en groupes de 10-20) sur le VIH et ont reçu une formation sur la façon dont le traitement ARV marche, ses effets secondaires habituels et comment les surmonter, l'importance de l'adhérence, et la résistance aux médicaments. Les infirmiers, les conseillers du dépistage volontaire, les médecins et les patients expérimentés participaient aux sessions sur l'adhérence. Plus récemment, ils ont aussi reçu une formation spécifique sur comment planifier les épisodes d'insécurité (décrit ci-dessous) et sur que faire en cas d' intensification du conflit causant une disruption du programme. Des réunions de groupe régulières ont eu lieu tout au long du traitement.

Dans chaque clinique, il y a  2 médecins, 4 infirmiers et quelques travailleurs volontaires ; le rapport médecin/patient est 1:500 pour le moment et continue d'augmenter.  Les responsabilités des médecins sont d'initier et de suivre le traitement ARV mais sont de plus en plus déléguées aux infirmiers. La clinique est soutenue par un laboratoire central, avec hématologie automatisée, biochimie et le comptage des CD4.  
 
Résultats du programme 

Entre mai 2002 et janvier 2006, 11,076 patients ont bénéficié du dépistage volontaire. Parmi eux, 19% étaient VIH positifs. De ceux testés VIH positifs, 94% (1868 patients) ont reçu un suivi dans une des cliniques VIH. (Figure 2)

En janvier 2006, le traitement ARV a été initié chez 494 (26%) patients. 66% d'entre eux étaient des femmes, avec un âge moyen de 37 ans (IQR 30-43) et un poids de 51 kg (IQR 44-58). A l'admission, 3%  étaient au stage clinique I de l'OMS, 12% au stage II , 49% au stage III  et 34% au stage IV; Le nombre médian de CD4 à l'admission était de 123 /mm3 (IQR 57-195). 95% des patients ont démontré une bonne adhérence au traitement ARV, défini comme manquant < 5% des doses à chaque visite, mesurée par le comptage des tablettes. 

La table 2 montre les résultats du traitement ARV comparés à d'autres cohortes de contextes à ressources limitées mais stables et de contextes riches[10]. Les augmentations en CD4 après 6 mois étaient similaires dans les 3 groupes, et les taux de mortalité et de perdus de vue à 12 mois sont favorables comparé au contexte stable à ressources limitées stable représenté dans la cohorte ART-LINC. Le taux d'adhérence rate, le gain en poids et CD4, les taux de mortalité et de perdus de vue sont tous comparés favorablement à ceux des programmes VIH des contextes stables à ressources limitées.[10-15] 

Conflit aigu dans la ville de Bukavu (Mai – Juin 2004) 

La plupart des conflits armés dans cette région sont survenus en dehors de Bukavu. Cependant, le 26 mai 2004, les force rebelles de l'armée congolaise envahirent la ville, entraînant une intensification du conflit pendant 13 jours. Durant cette période, des centaines de civils furent tués, des milliers ont fui au Rwanda, et de nombreuses femmes furent violées.[16]

Pendant cette crise, les mouvements du staff et des patients étaient réduits et tous les  expatriés MSF furent évacués de la ville. Les activités essentielles de la clinique VIH et de l'hôpital ont été maintenues pendant la plus grande partie de la durée du conflit par un infirmier congolais qui vivait à proximité, bien que la clinique restait inaccessible à beaucoup, en raison des distances et de l'insécurité. La communication durant cette période était difficile mais a continué grâce à l'émission radio et “bouche a oreille”. La pharmacie MSF n'a pas été pillée.

Avant le début de cette crise, 66 patients avaient commencé le traitement ARVet tels furent leurs résultats: 14 avaient reçu un approvisionnement suffisant en médicaments et n'ont pas du revenir à la clinique; 3 étaient hospitalisés et avaient donc accès aux médicaments; 41 sont venus pour leurs ARVs pendant la période de crise; 3 purent se procurer leurs ARVs au Rwanda en collaboration avec une autre mission MSF; 5 ont eu une interruption significative de leur traitement ARV (dont 4 avaient fui Bukavu). Néanmoins, tous les patients ont ré-établi le contact avec la clinique et ont repris leur traitement.


Huit mois après le conflit, une réunion de groupe a été organisée pour cinq des patients affectés et deux infirmiers. Ils furent sélectionnés par la simple méthode d'échantillonnage stratifié afin de représenter les effets d'être déplacé ou non, avec ou sans interruption de traitement. Les patients décrirent le stress physique et mental, la maladie, les distance géographiques, les limitations de mouvement, et la menace de violence nourriture manquante. Tous les patients étaient convaincus que leur vie dépendait de leur possibilité de rester adhérent au traitement ARV (Box 1).

Box 1 

Madame JEANNE 
Suivie à la clinique depuis 2002,sous ARV depuis décembre 2003. Vis au Rwanda 

Quand les luttes ont commencé, j'ai pensé que c'était la fin de nos vies. J'avais des ARV pour seulement 3 jours et 5 jours de sécurité. J'avais interrompu mon traitement pour 2 jours lorsque Sengi [un autre patient] est arrivé avec des médicaments envoyés par MSF. J'avais peur que ce soit la fin de ma vie, non seulement à cause du manque de médicaments, mais aussi parce que j'étais très malade et ai du être hospitalisée pour fièvre et vomissements. C'est très important d'avoir le traitement. 

Madame JULIE 
Suivie à la clinique depuis mai 2003, et sous ARV depuis Décembre 2003. 

J'ai entendu ces coups de feu durant toute la nuit. Lorsque je n'avais plus que 5 pilules, j'ai perdu mon appétit et me sentais désespérée… mais malgré l'incertitude, j'ai continué à prendre mon traitement à l'heure correcte… Quand je n'avais plus qu'une seule pilule, j'ai eu le courage d'aller chercher plus de traitement. Je suis allée voir l'infirmière Céline chez elle, qui m'a informée qu'elle pouvait me remettre des ARVs; Avec ça, j'avais de nouveau pour un mois de traitement. S'il nous avait fallu abandonner le traitement, on serait revenu au point de départ, très malade. 


Se préparer pour l'insécurité: L'importance du plan d'urgence
Notre expérience a montré qu'une des clefs du succès de l'approvisionnement en traitement ARV en situations de conflit est d'être prêt à affronter les interruptions. Les programmes doivent inclure un plan d'urgence qui permet au staff et aux patients de réagir le plus efficacement possible, même si le conflit s'intensifie. Les aspects essentiels d'un plan d'urgence sont: 

Education. L'éducation du patient doit souligner l'importance d'une adhérence rigoureuse au traitement ARV- en particulier, l'importance de ne pas réduire les doses de médicaments même si cela permet d'en avoir pour plus longtemps quand l'approvisionnement régulier est menacé. Néanmoins, les patients doivent comprendre qu'une interruption d'ARV inévitable, n'entraînera pas de résistance aux ARV, si elle est managée correctement. Ils doivent être formés à la prise en charge correcte (voir ci-dessous). Les patients doivent être informés sur le plan d'urgence durant les  périodes d'instabilité aiguë, qui comprend aussi les mécanismes d'approvisionnement et le support médical. 
 
Capacité en ressources humaines. L'ensemble du staff médical doit être bien formé aux bases de la prise en charge du VIH (y compris le traitement ARV) de manière à pouvoir remplir les trous en ressources humaines dus au conflit. Le but est de créer un staff polyvalent, capable de maintenir les activités de base si les médecins et autres sont évacués. 

Services de communication. Un service de communication entre le staff médical et les patients doit être établi à l'avance. Cela peut inclure des activités telles que des émissions radiodiffusées, des groupes d'église ou des posters. 

Stock de médicaments d'urgence et 'washout medications'. Un stock additionnel de 15 – 30 jours d 'urgence peut être remis à chaque patient. Ils doivent le ramener à chaque visite à la clinique afin de monitorer leur quantité et date d'expiration. Un 'washout' drug stock peut aussi être fourni. Les patients ayant un traitement contenant un Non-Nucleoside Reverse Transcriptase Inhibitor (NNRTI) peuvent être approvisionnés avec un 'wash-out' stock de 7 jours, qu'ils devraient prendre après la fin de leur traitement ARV habituel, quels que soient les NRTI utilisés dans leur traitement ( lamivudine and zidovudine, or stavudine and lamivudine). Ceci diminue les possibilités de résistance aux NNRTI pendant la période de monothérapie due à sa longue demi-vie.[17] 

Stock de sécurité. Les médicaments du VIH peuvent faire l'objet de pillage et vandalisme. Les stock de médicaments devraient être répartis et rangés dans différents endroits sécurisés, afin de minimiser l'impact d'éventuels pillages. La taille du stock au niveau du programme devrait être maintenue au minimum nécessaire dans les régions très instables.

Décentralisation des soins. La décentralisation des services cliniques à travers la région couverte permets de limiter le nombre de déplacements, ce qui assure un accès aux centres plus sûr et plus facile pour le staff et les patients en temps d'insécurité. 

Collaboration avec les centres de traitement VIH dans les régions voisines.  Des relations avec d'autres programmes, qui offrent traitement ARV dans les alentours, devraient être établies. Un accord d'aide mutuelle entre organisations peut être crucial dans le soin des patients déplacés ou lorsqu'un accès urgent à des médicaments est nécessaire en cas de vol ou destruction des stocks. 

Cartes de traitement informatives et données médicales en double. Tous les patients devraient avoir une carte d'identification personnelle contenant la liste de leurs ARV et autres informations médicales significatives, telles que les maladies associées, allergies, et le taux de CD4. Cette documentation est utile si le patient devient déplacé et cherche à obtenir son traitement ailleurs ou si les archives médicales de la clinique sont perdus ou détruits lors d'un pillage. Un double des dossiers médicaux devrait être rangé dans un endroit sûr hors de la clinique. 

Intégration avec d'autres services.  Un partenariat avec les services de santé gouvernementaux peut aider à assurer l'accès aux autorités et aux chaînes d'approvisionnement dans les situations de conflit aigu. Tout aussi importants, les liens de communication doivent être établis avec tous les intervenants du conflit. Ces relations peuvent aussi assurer quelque protection contre les pillages en temps de conflit. Idéalement, les bénéfices perçus du programme sont tels que la communauté entière, y compris le gouvernement, la société civile et les militaires, sont prêts à le supporter en dépit du conflit.

Discussion 

Le projet VIH de Bukavu montre que la mise en place des soins du VIH, y compris le traitement ARV, dans des régions de conflit chronique est faisable et  effective, avec des résultats précoces de traitement semblables à ceux de projets VIH des régions sans conflit. Ces résultats ont été atteints avec l'aide et les ressources de l'ONG internationale (MSF), dans un contexte urbain. NŽanmoins, les ŽlŽments clefs du plan d'urgence pour l'offre des soins en situation de conflit ne demandent pas de ressources supplŽmentaires et sont, semble-t-il, gŽnŽralement applicables ˆ tous les programmes de soins, supportŽs ou non par l'aide internationale.

Alors que l'instabilité en RDC a empêché la plupart des acteurs d'offrir le traitement ATV, le projet de Bukavu n'a connu qu'un épisode significatif d'interruption sur une pŽriode de 3 ans. Un aspect important tiré de notre expérience est que la définition de 'conflit chronique' ne veut pas nécessairement dire instabilité fréquente. En fait, le profil de conflit ˆ Bukavu s'est caractŽrisŽ par des pŽriodes de calme relatif, ponctuŽes par des Žpisodes d'insŽcuritŽ aigu‘, assez typique de nombreux conflits africains[18], et les le�ons apprises ˆ Bukavu sont actuellement appliquŽes dans d'autres projets MSF, affectŽs par un conflit.


Bien que les épisodes d'insécurité puissent déstabiliser les programmes ARV et soumettre les patients à un risque élevé d'interruption de traitement et de résistance aux médicaments, le plan d'urgence et le management des interruptions de traitement peuvent minimiser ces risques. En raison des incertitudes auxquelles sont soumis les programmes VIH dans des situations à ressources limitées – le risque de ruptures en médicaments dues à la mauvaise organisation, aux finances insuffisantes, au manque de staff – des éléments du plan d'urgence peuvent être aussi appropriés dans des programmes non affectés par un conflit.


Considérant les implications significatives entre conflit et VIH, il est particulièrement important de cibler les populations affectées par les 2 problèmes. En raison du nombre élevé de patients VIH positifs dans de nombreux pays affectés par un conflit, il n'est pas acceptable d'attendre que le conflit soit résolu avant d'offrir une réponse.

Acknowledgements 

We thank the MSF team for their hard work during and after the events described in this document, with specific thanks to Dr Rebecca Adlington, and Dr Leslie Shanks. Thanks also must go to Dr Victorio Torres-Feced, MSF AIDS advisor at the time of this work, for his encouragement in proceeding with the development of this document. 
Ethics statement: ethics approval for the use of patient data and patient testimonials was granted by the Université Catholique de Bukavu, Commission Institutionnelle d'éthique on May 25, 2005.

References 

1.	Uppsala Conflict Data Program. Uppsala conflict database, 2004. Accessed 2005. http://www.pcr.uu.se/database/index.php.
2.	WHO UNAIDS. Aids epidemic update: December 2005. In. Geneva: UNAIDS and WHO; 2005:1-90.
3.	Mock NB, Duale S, Brown LF, Mathys E, O'Maonaigh H C, et al. Conflict and HIV: A framework for risk assessment to prevent HIV in conflict-affected settings in Africa. Emerg Themes Epidemiol 2004,1:6.
4.	Hankins CA, Friedman SR, Zafar T, Strathdee SA. Transmission and prevention of HIV and sexually transmitted infections in war settings: implications for current and future armed conflicts. Aids 2002,16:2245-2252.
5.	Ellman T, Culbert H, Torres-Feced V. Treatment of AIDS in conflict-affected settings: a failure of imagination. Lancet 2005,365:278-280.
6.	Spiegel PB. HIV/AIDS among conflict-affected and displaced populations: dispelling myths and taking action. Disasters 2004,28:322-339.
7.	Coghlan B, Brennan RJ, Ngoy P, Dofara D, Otto B, et al. Mortality in the Democratic Republic of Congo: a nationwide survey. Lancet 2006,367:44-51.
8.	International Crisis Group. Africa report N 34: A Congo Action Plan. 19 October, 2005.
9.	World Health Organisation. Scaling up antiretroviral therapy in resource-limited settings. Treatment guidelines for a public health approach (revision). Geneva; World Health Organisation, 2004. QV268.5, 2004.
10.	Braitstein P, Brinkhof MW, Dabis F, Schechter M, Boulle A, et al. Mortality of HIV-1-infected patients in the first year of antiretroviral therapy: comparison between low-income and high-income countries. Lancet 2006,367:817-824.
11.	Calmy A, Pinoges L, Szumilin E, Zachariah R, Ford N, et al. Generic fixed-dose combination antiretroviral treatment in resource-poor settings: multicentric observational cohort. Aids 2006,20:1163-1169.
12.	Ferradini L, Jeannin A, Pinoges L, Izopet J, Odhiambo D, et al. Scaling up of highly active antiretroviral therapy in a rural district of Malawi: an effectiveness assessment. Lancet 2006,367:1335-1342.
13.	Tassie JM, Szumilin E, Calmy A, Goemaere E. Highly active antiretroviral therapy in resource-poor settings: the experience of Medecins Sans Frontieres. Aids 2003,17:1995-1997.
14.	Akileswaran C, Lurie MN, Flanigan TP, Mayer KH. Lessons learned from use of highly active antiretroviral therapy in Africa. Clin Infect Dis 2005,41:376-385.
15.	Etard JF, Ndiaye I, Thierry-Mieg M, Gueye NF, Gueye PM, et al. Mortality and causes of death in adults receiving highly active antiretroviral therapy in Senegal: a 7-year cohort study. Aids 2006,20:1181-1189.
16.	International CrisisGroup. Africa Report N 21: Back to the brink in the Congo. 17 December, 2004.
17.	Chaix ML, Ekouevi DK, Rouet F, Tonwe-Gold B, Viho I, et al. Low risk of nevirapine resistance mutations in the prevention of mother-to-child transmission of HIV-1: Agence Nationale de Recherches sur le SIDA Ditrame Plus, Abidjan, Cote d'Ivoire. J Infect Dis 2006,193:482-487.
18.	Human Security Centre, University of British Columbia, Canada.  Human Security Report 2005.  Oxford University Press, 2005.


Table 1. Obstacles to providing HIV care in resource limited chronic conflict settings

Medical System related Obstacles	Ø	Limited (or absent) Health Infrastructure 
Ø	Limited (or absent) Human resources (persons and skills)
Ø	Limited financial resources
Ø	Limited political leadership, will, coordination or competency
	
Population related Obstacles	Ø	Poverty
Ø	Stigma
Ø	Limited HIV awareness/understanding
	
Conflict related Obstacles 	Ø	Instability of Security 
l	Interruption in  program continuity/safety
Ø	Instability of Populations 
l	Increased migration or population movement
	

Table 2.: Comparison of ART patient outcomes comparing programmes in chronic conflict with non-conflict settings in low income and high income settings. 
	Bukavu
(Chronic Conflict Setting)	ART-LINC [10]
(Low Income Setting)	ART-CC [10]
(High Income Setting)	
No. of patients initiating ART 	494	4810	22 217	
No. of patient years on therapy (Median duration on ART)	235	3744	24 310	
Baseline Median CD4 count	123
[IQR 57 – 195]	108
[IQR 37 – 210]	234
[IQR 98 - 380]	
6 month median weight gain (kg) [IQR]	2.5[0-5.5]	-	-	
6 month Median CD4 gain (cells/ml) [IQR]	163
[IQR 82 – 232]	106
[IQR 43 – 180]	103
[IQR 32 - 192]	
12 month Mortality	7.9%
(95% CI 3.6–12.1)	6.4%
(95% CI 5.1 – 7.7)	1.8%
(95% CI 1.5 – 2.2)	
12 month Loss to follow-up	5.4%
(95% CI 3.2-7.5)	15% 
(No CI given)	5% 
(No CI given)	


Figure 1: Map Showing Bukavu, South Kivu, DRC (Figure: Anthony Flores)
Figure 2.  Monthly number of people undergoing VCT, testing HIV positive, and enrolling in the HIV clinic
