# Supplementary material for: HIV Treatment in a Conflict Setting: Outcomes and Experiences from Bukavu, Democratic Republic of the Congo
Source: PLoS Med. 2007 May 29;4(5):e129. doi: 10.1371/journal.pmed.0040129 (PMC1880839; doi:10.1371/journal.pmed.0040129)
Supplement: Alternative Language Text S3 — (31 KB DOC). [file pmed.0040129.sd003.doc]

**SALUD EN ACCIÓN**

**Tratamiento del VIH en un marco de conflicto:**

**Resultados y experiencias desde Bukavu, República Democrática del Congo**

**Heather Culbert^1^, David Tu^1^, Daniel P. O’Brien^1^, Tom Ellman^2^, Clair Mills^1^, Nathan Ford^2^, Tina Amisi^3^, Keith Chan^4^, Sarah Venis^2^**

1 Medecins Sans Frontieres Holanda, Amsterdam

2 Medecins Sans Frontieres Reino Unido, Londres

3 Medecins Sans Frontieres Holanda, Bukavu, República Democrática del Congo.

4 British Columbia Center for Excellence for HIV/AIDS, Vancouver, Canadá.

Los conflictos armados y la infección por VIH han ejercido un profundo impacto en las sociedades de África subsahariana. El número de países implicados en algún conflicto armado ha fluctuado, llegando a un total de 24 estados africanos en 2004[1]; la mayoría de estos conflictos son intraestatales y crónicos. La región también alberga la mayor tasa de infección por VIH del mundo, con más de 25 millones de casos documentados de personas infectadas.[2]

Aunque la interrelación entre VIH y conflicto está cada vez más clara,[3-6] el efecto del conflicto y la inestabilidad sobre la incidencia y prevalencia del VIH puede ser impredecible.[6] Gran parte de los estudios sobre riesgos son poco fiables, y un conflicto puede tanto proteger una población del VIH aislando comunidades de la propagación de la infección, como aumentar el riesgo de un individuo por medio del desplazamiento, la violencia sexual, y la desestructuración de comunidades e instituciones sanitarias.

A pesar de las a menudo significativas tasas de mortalidad y morbilidad relacionadas con el VIH, pocas veces se han intentado llevar a cabo programas de atención al VIH en zonas de conflicto. Existe la percepción de que resultaría demasiado difícil realizarlos de manera segura y efectiva, y que la prevención y el tratamiento contra el VIH son secundarios a problemas tales como la alimentación, la vivienda, el agua y la sanidad, la asistencia médica básica, y la seguridad personal. Como se resume en la Tabla 1, proporcionar atención al VIH en un escenario de conflicto implica obstáculos adicionales a los que generalmente se encuentran en otros marcos de recursos limitados.

Este artículo describe lo que se ha aprendido a través de una experiencia de 3 años proporcionando asistencia al VIH, incluyendo terapia antirretroviral (TAR), a una población afectada por el conflicto en la República Democrática del Congo (RDC).
